# Association between urogenital inflammation conditions and leukocytospermia: insights from a large-scale cross-sectional study

**DOI:** 10.3389/fcimb.2026.1890531

**Published:** 2026-08-12

**Authors:** Nan Guo, Zeling Zhang, Ming Li, Qinyi Zhang, Shun Bai, Xiaojin He

**Affiliations:** 1 Department of Obstetrics and Gynecology, The First Affiliated Hospital of Anhui Medical University, Hefei, China; 2 Center for Reproduction and Genetics, Department of Obstetrics and Gynecology, The First Affiliated Hospital of USTC, Division of Life Sciences and Medicine, University of Science and Technology of China, Hefei, China; 3 Reproductive Medicine Center, The First Affiliated Hospital of Anhui University of Chinese Medicine, Hefei, Anhui, China; 4 School of Medicine, Anhui University of Science and Technology, Huainan, China; 5 NHC Key Laboratory of Study on Abnormal Gametes and Reproductive Tract, Anhui Medical University, Hefei, China; 6 Reproductive Medicine Center, Department of Obstetrics and Gynecology, Shanghai General Hospital, Shanghai Jiao Tong University School of Medicine, Shanghai, China

**Keywords:** epididymitis, leukocytospermia, prostatitis, seminal leukocytes, urogenital inflammation conditions

## Abstract

**Background:**

Leukocytospermia (LCS) is commonly identified during male infertility evaluation and is associated with impaired semen quality. However, the specific roles of various inflammatory conditions in LCS remain incompletely understood.

**Methods:**

This cross-sectional study enrolled 9345 males who attended an andrology clinic during the period from April 2020 to March 2023. Demographic and lifestyle factors, medical history, and clinically defined urogenital inflammatory conditions (epididymitis, prostatitis, orchitis, and seminal vesiculitis) were obtained from questionnaires and clinical records. LCS was defined as >1 × 10^6^ peroxidase-positive leukocytes/mL of semen. Multivariable logistic regression models were used to assess associations with LCS and to estimate odds ratios (ORs) and 95% confidence intervals (CIs).

**Results:**

Of the 9345 men, 1068 (11.4%) had LCS. Men with LCS were slightly older and were more often smokers. Epididymitis (1.69% vs 0.85%, P = 0.007) and prostatitis (14.42% vs 10.61%, P < 0.001) were more common in men with LCS. Men with LCS also had lower semen volume, sperm concentration, progressive motility, total motility, total motile sperm count, and normal morphology, and higher sperm DNA fragmentation index and high DNA stainability. In the fully adjusted model, epididymitis (aOR = 1.88, 95% CI 1.10-3.21) and prostatitis (aOR = 1.37, 95% CI 1.14-1.66) were associated with higher odds of LCS. Estimates for orchitis and seminal vesiculitis were imprecise because these conditions were uncommon.

**Conclusions:**

Epididymitis and prostatitis were associated with higher odds of LCS in men attending an andrology clinic. Our study findings support assessment of urogenital inflammatory conditions in men with LCS.

## Introduction

Male infertility is a common clinical challenge, and semen analysis remains central to the evaluation of male reproductive function. Leukocytospermia, characterized as the presence of more than one million leukocytes per milliliter of semen by World Health Organization (WHO) guidelines, is usually linked with male infertility. Clinical studies have reported that approximately 10-30% of infertile men exhibit leukocytospermia (Bai et al., 2021; Assidi, 2022; Rivero et al., 2023). The presence of elevated leukocyte levels in semen is associated with a decrease in semen quality (Fan et al., 2023). Leukocytes in the semen, particularly neutrophils, release a variety of enzymes, cytokines, and reactive oxygen species (ROS) that can damage sperm DNA, and mitochondria (Fariello et al., 2009). This damage can lead to reduced sperm motility, abnormal morphology, and decreased sperm viability (Fraczek et al., 2016). Additionally, studies have shown that men with high semen leukocyte counts tend to have lower fertilization rates in *in vitro* fertilization (IVF) and intracytoplasmic sperm injection (ICSI) procedures (Sukcharoen et al., 1995; Moilanen et al., 1998).

Urogenital infection and inflammation are related but are not synonymous. Sexually transmitted and urinary pathogens, including *Chlamydia trachomatis*, *Neisseria gonorrhoeae*, and enteric bacteria, may cause inflammatory conditions such as epididymitis or bacterial prostatitis (Bai et al., 2022). However, male genital tract inflammation may also occur without microbiologically confirmed infection. The relationship between urogenital tract inflammation (e.g., epididymitis, prostatitis, and urethritis) and leukocytes in semen is a critical area of study in male infertility (Sharma et al., 2022; Fan et al., 2023). Inflammatory stimuli can recruit and activate leukocytes in the male genital tract and increase the number of peroxidase-positive leukocytes in semen. Once the concentration exceeds the WHO threshold, LCS is present. Inflammatory processes lead to the release of ROS and pro-inflammatory cytokines (e.g., TNF-α, IL-6, IL-8) by leukocytes, which impair sperm membrane integrity, motility, and DNA quality (Haidl et al., 2015). Common inflammatory conditions, such as prostatitis (prevalence of 4% to 11% in men under 50) and epididymitis, can spread to the testis as ‘epididymo-orchitis’, leading to spermatogenic impairment, testicular atrophy, or even arrest of spermatogenesis (Nickel et al., 2001; Choi et al., 2020). These inflammatory conditions are strongly linked to male infertility, with studies indicating that 15% of infertile men exhibit signs of reproductive tract infection (Pellati et al., 2008). Since research has established a link between urogenital tract inflammation and leukocyte levels in semen, studies on the correlation between different types of urogenital inflammation and leukocytospermia remain unknown.

Age, smoking, chronic systemic disease, and other clinical factors may also be associated with seminal leukocyte concentrations (Ilacqua et al., 2018; Dutta et al., 2025; Gill et al., 2024). The aim of this study was therefore to assess the associations between clinically defined epididymitis, prostatitis, orchitis, and seminal vesiculitis and LCS in men attending an andrology clinic. We also compared semen parameters between men with and without LCS.

## Methods

### Participants

**Figure 1:**
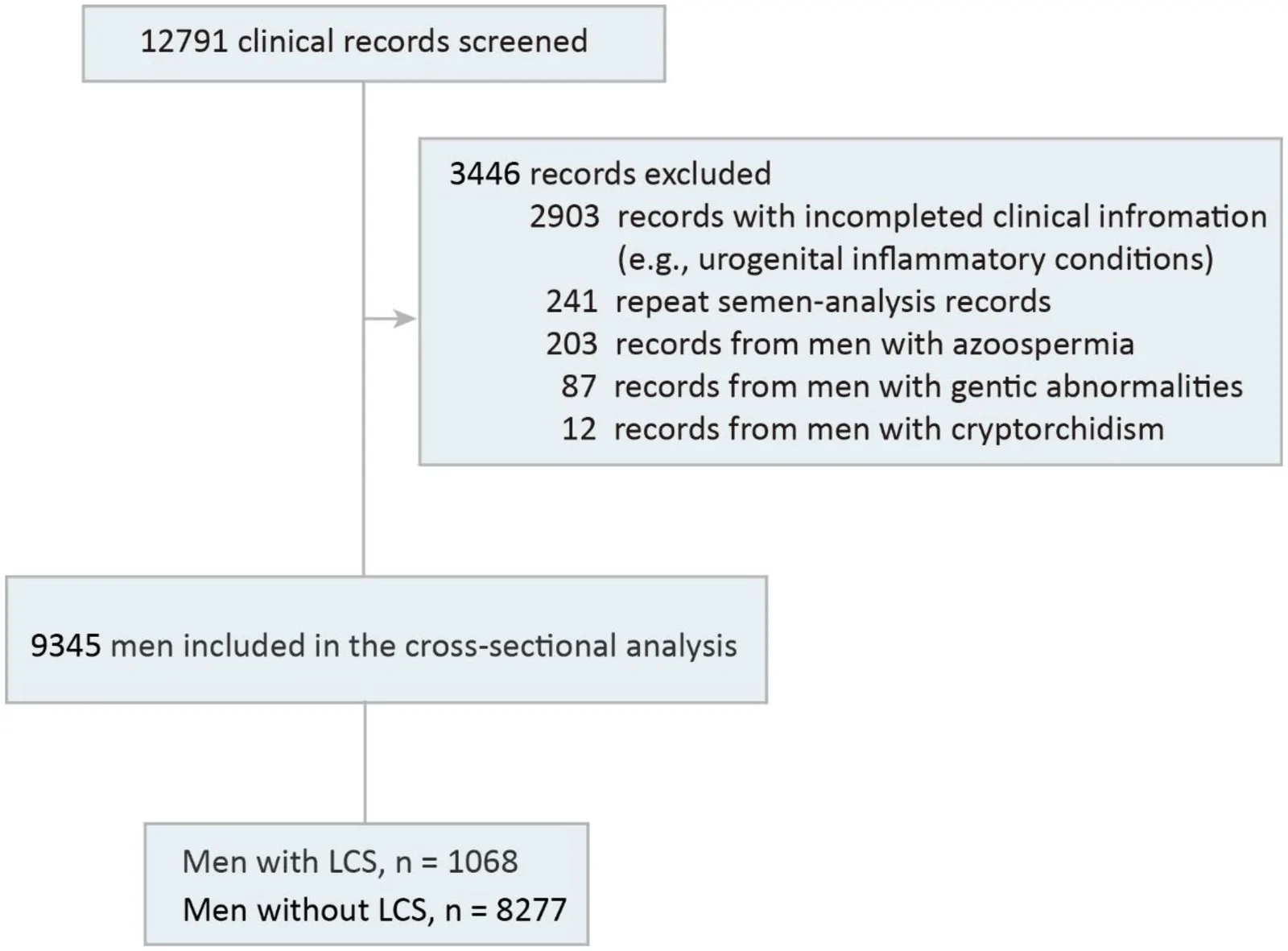
Flow diagram of the study. LCS, leukocytospermia.

This is a cross-sectional study included 12,791 clinical records with semen analysis data from men attending the andrology clinic between April 2020 and March 2023 (**Figure 1**). We excluded 2903 records because of incomplete clinical information, 241 repeated semen-analysis records, 203 records from men with azoospermia, 87 records from men with genetic abnormalities, and 12 records from men with cryptorchidism. The final analytical sample comprised 9345 men, with one semen-analysis record and one questionnaire per participant.

The inclusion criteria were as follows: (1) 18 years of age or older and (2) no antibiotic treatment within the preceding two weeks. At the clinical visit, participants completed a standardized questionnaire covering smoking, alcohol consumption, educational level, sexual frequency, duration of infertility, and medical history. Clinical information, including chronic systemic disease, varicocele, redundant foreskin, cryptorchidism, and previous genital surgery, was obtained from the questionnaire and clinical records. The study protocol was approved by the Institutional Review Board (Approval No. 2023-RE-196) and is reported according to the Strengthening the Reporting of Observational Studies in Epidemiology (STROBE) statement.

Urogenital inflammatory conditions included chronic epididymitis, prostatitis, orchitis, and seminal vesiculitis. Chronic epididymitis was defined by unilateral or bilateral epididymal pain or discomfort for the previous 3 months together with induration, irregularity, or thickening on clinical examination. Prostatitis was defined by persistent pain in the prostate region at least three months, with urinary and/or sexual symptoms during the previous 3 months. Orchitis was identified by unilateral or bilateral testicular swelling, atrophy, or pain, with fever or scrotal erythema during the previous 3 months. Seminal vesiculitis was identified by hematospermia, painful ejaculation, and perineal or lower abdominal discomfort during the previous 3 months.

### Semen analysis

Semen samples were collected via masturbation after abstinence of 2–7 days. All analyses were performed in accordance with the fifth edition of the WHO Laboratory Manual for the Examination and Processing of Human Semen (World Health Organization, 2010). After collection, each sample was placed in a 37 °C incubator for ≥30 min to ensure complete liquefaction before any manipulation. Sperm concentration, progressive motility, total motility, and detailed kinematic variables were quantified using a computer-assisted sperm analyzer (CASA; SAS-II, SAS Medical, Beijing, China) that had been calibrated with latex beads of known concentration and motility. The total motile sperm count (TMSC) was computed as: TMSC = ejaculate volume (mL) × sperm concentration (×10^6^/mL) × progressive motility (%). Sperm morphology was assessed after Diff-Quik staining (Anke Biotechnology, Hefei, China). A minimum of 200 spermatozoa per specimen were examined at 1 000× magnification under oil immersion. Leukocyte quantification in seminal plasma was carried out by peroxidase staining with benzidine (Anke Biotechnology) and expressed as ×10^6^ cells/mL. The presence of anti-sperm antibodies (AsAs) was determined by the mixed antiglobulin reaction (MAR) test (Anke Biotechnology) following the manufacturer’s protocol. The sperm DNA fragmentation index (DFI) and high DNA stainability (HDS) were evaluated by the sperm chromatin structure assay (SCSA; Celula, Chengdu, China) on an Accuri C6 flow cytometer (BD Biosciences). All technicians received standardized training in semen collection, handling, and analysis procedures provided by the National Research Institute for Family Planning. The laboratory participates continuously in the External Quality Assessment (EQA) program coordinated by the NHC Key Laboratory of Male Reproduction and Genetics.

### Statistical analysis

Categorical variables are expressed as counts and proportions, whereas continuous variables are presented as the mean ± standard deviation (SD) when normally distributed or as the median with interquartile range (IQR) otherwise. Normality was assessed with the Shapiro-Wilk test. Between-group comparisons of categorical data were performed with Pearson’s chi-square test. Differences in continuous variables were evaluated using the two-sample Student t test (parametric) or the Mann-Whitney U test (non-parametric), as appropriate. Logistic regression models were used to assess associations with LCS and to estimate ORs and 95% CIs. Covariates were selected *a priori* based on clinical knowledge and the directed acyclic graph (DAG, **Supplementary Figure 1**). Model 1 adjusted for age, BMI, educational level, smoking, and alcohol consumption. Model 2 was an extended clinical model and additionally adjusted for sexual frequency, duration of infertility, chronic systemic disease, redundant foreskin, and varicocele. Model 1 was used for primary analyses; Model 2 was used for supplementary purposes to assess the robustness of the estimates in a more comprehensively adjusted clinical context. Given the cross-sectional design and uncertainty regarding the causal roles of chronic systemic diseases, results from Model 2 should be interpreted with caution. Additional analyses stratified by clinically relevant subgroups (age <30 vs ≥30 years; BMI <24 vs ≥24 kg/m²; education level; smoking status; alcohol consumption; infertility duration; sexual frequency; chronic disease; varicocele; and redundant foreskin) were undertaken to explore potential effect modification. Interaction P values were not adjusted for multiple testing and were interpreted cautiously. Because orchitis and seminal vesiculitis were uncommon, these conditions were included in descriptive and crude analyses but were not entered into the fully adjusted condition-specific models. All analyses were two-tailed, and statistical significance was set at *P* < 0.05. Data management and statistical computations were executed in R version 4.3.3 (R Foundation for Statistical Computing, Vienna, Austria).

## Results

Of the 9345 participants included in the analysis, 1068 were in the LCS group and 8277 were in the non-LCS group (**Table 1**). The mean age of the participants was significantly higher in the LCS group (31.4 years ± 5.1) compared to the non-LCS group (30.7 years ± 4.9; *P* < 0.001). There was no significant difference in BMI between the LCS group (24.6 kg/m² ± 3.7) and the non-LCS group (24.6 kg/m² ± 3.5; *P* = 0.99). Smoking status differed significantly between the groups, with a higher proportion of smokers in the LCS group (49.3%) compared with the non-LCS group (43.6%; *P* < 0.001). No significant differences were found for alcohol consumption, educational level, sexual frequency, duration of infertility, varicocele, or redundant foreskin. The prevalence of chronic diseases was slightly higher in the LCS group (12.0%) compared to the non-LCS group (10.1%; *P* = 0.05). Epididymitis was more common in the LCS group (1.69%) compared to the non-LCS group (0.85%; *P* = 0.007). Similarly, prostatitis was more common in the LCS group (14.42%) compared to the non-LCS group (10.61%; *P* < 0.001). No significant differences were found in the prevalence of orchitis (*P* = 0.33) or seminal vesiculitis (*P* = 0.64).

**Table 1:** Clinical characteristics of the 9345 subjects included in this study.

Clinical characteristics	Total (n=9345)	Non-LCS (n=8277)	LCS (n=1068)	*P*
Age (years), mean ± SD	30.8 ± 5.0	30.7 ± 4.9	31.4 ± 5.1	< 0.001
BMI (kg/m^2^), mean ± SD	24.6 ± 3.5	24.6 ± 3.5	24.6 ± 3.7	0.99
Lifestyle factors				
Smoking^a^, n (%)				< 0.001
Nonsmoker	5209(55.7)	4668(56.4)	541(50.7)	
Smoker	4136(44.3)	3609(43.6)	527(49.3)	
Drinking^b^, n (%)				0.95
Nondrinker	4339(46.4)	3844(46.4)	495(46.3)	
Drinker	5006(53.6)	4433(53.6)	573(53.7)	
Education, n (%)				0.16
Middle school or lower^c^	2051(21.9)	1804(21.8)	247(23.1)	
High school	1672(17.9)	1465(17.7)	207(19.4)	
College	5622(60.2)	5008(60.5)	614(57.5)	
Sex frequency (weekly), n (%)				0.42
< 1	1321 (14.1)	1180 (14.3)	141 (13.2)	
1-2	5857(62.7)	5192(62.7)	665(62.3)	
> 2	2167 (23.2)	1905 (23.0)	262 (24.5)	
Medical history				
Duration of infertility (year), n(%)				0.89
< 1	4708 (50.4)	4172 (50.4)	536 (50.2)	
≥ 1	4637 (49.6)	4105 (49.6)	532 (49.8)	
Chronic diseases^d^, n (%) No Yes	8384(89.7) 961(10.3)	7444(89.9) 833(10.1)	940(88.0) 128(12.0)	0.05
Varicocele, n (%)				0.39
No	8728(93.4)	7724(93.3)	1004(94.0)	
Yes	617(6.6)	553(6.7)	64(6.0)	
Redundant foreskin, n (%)				0.22
No	6473(69.3)	5716(69.1)	757(70.9)	
Yes	2872(30.7)	2561(30.9)	311(29.1)	
Urogenital inflammation conditions				
Epididymitis, n(%)				0.007
No	9257 (99.1)	8207 (99.2)	1050 (98.3)	
Yes	88 (0.9)	70 (0.8)	18 (1.7)	
Prostatitis, n(%)				<0.001
No	8313 (89.0)	7399 (89.4)	914 (85.6)	
Yes	1032 (11.0)	878 (10.6)	154 (14.4)	
Orchitis, n(%)				0.33
No	9287 (99.4)	8228 (99.4)	1059 (99.2)	
Yes	58 (0.62)	49 (0.6)	9 (0.8)	
Seminal vesiculitis, n(%)				0.64
No	9305 (99.6)	8243 (99.6)	1062 (99.4)	
Yes	40 (0.4)	34 (0.4)	6 (0.6)	

BMI, body mass index; SD, standard deviation.

P-values were calculated using the Student’s t-test, Kruskal-Wallis rank sum test or chi-square test.

^a^ included never smoking and no smoking during the past 3 months.

^b^ included never drinking and no drinking during the past 3 months.

^c^ included primary school and junior high school.

^d^ included diabetes, hypertension and dyslipidemia.

The semen parameters are also shown in **Table 2**. Compared with men without LCS, men with LCS had lower semen volume (2.8 vs 3.1 mL; *P* < 0.001), sperm concentration (56.7 vs 62.0 million/mL; P = 0.02), progressive motility (35.9% vs 38.1%; *P* < 0.001), total motility (41.0% vs 43.3%; *P* < 0.001), TMSC (55.2 vs 69.4 million; *P* < 0.001), and normal morphology (6.0% vs 7.0%; *P* = 0.01). The median concentration of leukocytes in semen was found to be higher in the LCS group than in the non-LCS group (2.3 × 10^6^ cells/mL versus 0.1 × 10^6^ cells/mL, *P* < 0.001). Men with LCS also had slightly lower sperm viability (72.0% vs 73.0%; *P* = 0.01) and higher DFI (15.2% vs 12.6%; *P* < 0.001) and HDS (7.6% vs 6.6%; *P* < 0.001). No significant difference was observed in AsAs (*P* = 0.14).

**Table 2:** Semen parameters of the 9345 subjects included in this study.

Semen parameters	Total (n=9345)	Non-LCS (n=8277)	LCS (n=1068)	*P*
Volume (mL), median (IQR)	3.1 (2.2-4.2)	3.1 (2.2-4.2)	2.8 (2.0-3.9)	<0.001
Concentration (million/mL), median (IQR)	61.4 (31.1-110.9)	62.0 (31.5-111.3)	56.7 (27.6-105.4)	0.02
Progressive motility (%), median (IQR)	37.8 (24.5-51.5)	38.1 (24.8-51.8)	35.9 (22.6-48.8)	<0.001
Total motility (%), median (IQR)	43.0 (28.1-57.9)	43.3 (28.4-58.2)	41.0 (26.5-55.6)	<0.001
TMSC (million), median (IQR)	67.5 (22.9-150.9)	69.4 (23.9-154.2)	55.2 (16.5-125.7)	<0.001
Normal morphology (%), median (IQR; n)	7.0 (5.0-8.0; 9255)	7.0 (5.0-8.0; 8208)	6.0 (4.0-8.0; 1047)	0.01
Leukocytes(×10^6^/mL), median (IQR)	0.1 (0-0.3)	0.1 (0.0-0.2)	2.3 (1.4-4.6)	<0.001
AsAs (%), median (IQR; n)	1.0 (0-3.0; 6579)	1.0 (0.0-3.0; 5800)	1.0 (0.0-3.0; 779)	0.14
Viability (%), median (IQR; n)	73.0 (62.0-83.0; 3909)	73.0 (62.0-84.0; 3441)	72.0 (60.0-80.0; 468)	0.01
DFI (%), median (IQR; n)	12.9 (8.3-19.7; 2183)	12.6 (8.2-19.4; 1941)	15.2 (9.6-23.8; 242)	<0.001
HDS (%), median (IQR)	6.7 (5.0-9.0; 2183)	6.6 (4.9-8.8; 1941)	7.6 (5.8-10.8; 242)	<0.001

IQR, interquartile range; TMSC, total motile sperm count; AsAs, antisperm antibodies; DFI, DNA fragmentation index; HDS, high DNA stainability.

Logistic regression results are shown in **Table 3**. Each 1-year increase in age was associated with 2% higher odds of LCS (OR = 1.02, 95% CI: 1.01-1.04; *P* < 0.001), and smokers had higher odds of LCS than nonsmokers (OR = 1.26, 95% CI: 1.11-1.43; *P* < 0.001). A significant association was observed between urogenital inflammatory condition and LCS (OR = 1.48, 95% CI: 1.24-1.77; *P* < 0.001). Epididymitis (OR = 2.01, 95% CI: 1.19-3.39; *P* = 0.009), and prostatitis (OR = 1.42, 95% CI: 1.18-1.71; *P* < 0.001) were found to be independently associated with higher odds of LCS. No significant associations were found for BMI, alcohol consumption, educational level, sexual frequency, duration of infertility, varicocele, or redundant foreskin. The crude estimates for orchitis (OR = 1.43, 95% CI 0.70-2.91) and seminal vesiculitis (OR = 1.37, 95% CI 0.57-3.27) were imprecise.

**Table 3:** Logistic regression analyses for the relationship between clinical characteristics and LCS.

Variables	β	SE	Z	OR (95% CI)	*P*
Age	0.02	0.01	3.80	1.02 (1.01-1.04)	< 0.001
BMI	-0.00	0.01	-0.00	1.00 (0.98-1.02)	0.99
Smoking					
Nonsmoker				1.00 (Reference)	
Smoker	0.23	0.07	3.55	1.26 (1.11-1.43)	< 0.001
Drinking					
Nondrinker				1.00 (Reference)	
Drinker	0.00	0.07	0.06	1.00 (0.88-1.14)	0.95
Education					
Middle school or lower				1.00 (Reference)	
High school	0.03	0.10	0.31	1.03 (0.85-1.26)	0.75
College	-0.11	0.08	-1.38	0.90 (0.77-1.05)	0.17
Sex frequency (weekly)					
< 1				1.00 (Reference)	
1-2	0.07	0.10	0.71	1.07 (0.88-1.30)	0.48
> 2	0.14	0.11	1.27	1.15 (0.93-1.43)	0.20
Duration of infertility (year)					
< 1				1.00 (Reference)	
≥ 1	0.01	0.07	0.13	1.01 (0.89-1.15)	0.89
Chronic diseases					
No				1.00 (Reference)	
Yes	0.20	0.10	1.94	1.22 (1.00-1.48)	0.05
Varicocele					
No				1.00 (Reference)	
Yes	-0.12	0.14	-0.85	0.89 (0.68-1.16)	0.39
Redundant foreskin					
No				1.00 (Reference)	
Yes	-0.09	0.07	-1.21	0.92 (0.80-1.05)	0.23
Urogenital inflammation conditions					
No				1.00 (Reference)	
Yes	0.39	0.09	4.40	1.48 (1.24-1.77)	< 0.001
Epididymitis					
No				1.00 (Reference)	
Yes	0.70	0.27	2.62	2.01 (1.19-3.39)	0.009
Prostatitis					
No				1.00 (Reference)	
Yes	0.35	0.09	3.72	1.42 (1.18-1.71)	< 0.001
Orchitis					
No				1.00 (Reference)	
Yes	0.36	0.36	0.98	1.43 (0.70-2.91)	0.33
Seminal vesiculitis					
No				1.00 (Reference)	
Yes	0.31	0.44	0.71	1.37 (0.57-3.27)	0.48

OR, Odds ratio; CI, Confidence interval.

Exploratory subgroup analyses were performed for epididymitis and prostatitis (**Supplementary Figure 2**). The overall associations were observed for both epididymitis (OR = 1.94, 95% CI 1.14-3.29; *P* = 0.014) and prostatitis (OR = 1.39, 95% CI 1.15-1.67; *P* < 0.001). No strong evidence of interaction was observed across the prespecified subgroups.

Multivariable logistic regression analyses revealed significant associations between specific urogenital inflammatory conditions and the likelihood of LCS (**Table 4**). For epididymitis, the aOR was 1.82 (95% CI 1.07-3.09; *P* = 0.03) in Model 1 and 1.88 (95% CI 1.10-3.21; *P* = 0.02) in Model 2. For prostatitis, the corresponding aORs were 1.37 (95% CI 1.13-1.65; *P* = 0.001) and 1.37 (95% CI 1.14-1.66; *P* < 0.001), respectively.

**Table 4:** The association among epididymitis, prostatitis and LCS in 9345 males.

Urogenital inflammation conditions	Non-adjusted		Adjusted I		Adjusted II	
	OR (95% CI)	*P*	aOR (95% CI)	*P*	aOR (95% CI)	*P*
Epididymitis						
No	1.00 (Reference)		1.00 (Reference)		1.00 (Reference)	
Yes	2.01 (1.19-3.39)	0.009	1.82 (1.07-3.09)	0.03	1.88 (1.10-3.21)	0.02
Prostatitis						
No	1.00 (Reference)		1.00 (Reference)		1.00 (Reference)	
Yes	1.42 (1.18-1.71)	< 0.001	1.37 (1.13-1.65)	0.001	1.37 (1.14-1.66)	< 0.001

OR, Odds ratio; CI, Confidence interval; aOR, adjusted OR.

Adjusted I, adjusted age, BMI, smoking, alcohol consumption, education.

Adjusted II, adjusted age, BMI, smoking, alcohol consumption, education, sex frequency, duration of infertility, the presence of chronic diseases, redundant foreskin and varicocele.

## Discussion

In this cross-sectional study of 9345 men attending an andrology clinic, we found that epididymitis and prostatitis were more common in men with LCS and were associated with higher odds of LCS after multivariable adjustment. Men with LCS were also slightly older, were more often smokers, and had lower semen volume, sperm concentration, progressive and total motility, TMSC, and normal morphology, together with higher DFI and HDS. Orchitis and seminal vesiculitis were uncommon, and the estimates for these conditions were imprecise. These findings describe cross-sectional associations and do not establish that the inflammatory conditions preceded LCS.

Previous studies have shown the correlation between demographic factors and lifestyle habits with leukocytospermia (Dutta et al., 2025). A study by Gill P. et al. included a total of 5435 IVF cycles for analysis and found that the LCS group had a significantly higher average age of 37.3 years, compared to 36.9 years in the non-LCS group (*P* = 0.004) (Gill et al., 2024). Similarly, our study also indicates that the leukocytospermia group being slightly older (LCS vs non-LCS, 31.4 vs 30.7). However, the absolute difference in our study was modest, and most participants were young men of reproductive age. Our data do not support a specific age-related immune mechanism. The proportion of smokers was also higher in the LCS group. A previous study in infertile men reported higher seminal leukocyte counts among cigarette smokers (Zhang et al., 2013). Cigarette smoke contains oxidant and toxic compounds, including polycyclic aromatic hydrocarbons and heavy metals, which may promote oxidative and inflammatory responses (Ilacqua et al., 2018). These compounds and inflammatory mediators were not measured in our study, and a biological mechanism cannot be inferred from the present data.

Prostatitis is a heterogeneous clinical syndrome, and bacterial prostatitis should be distinguished from chronic pelvic pain or inflammatory conditions without proven bacterial infection (Brunner et al., 1983; Nickel et al., 2001). The effect of prostatitis on conventional semen parameters remains controversial (Alshahrani et al., 2013). Several studies reported that chronic prostatitis negatively impact on semen quality (Menkveld et al., 2003; Marconi et al., 2009). However, other studies have failed to show significant differences in semen parameters between individuals with prostatitis and control groups (Pasqualotto et al., 2000; Ludwig et al., 2003). Indeed, Motrich et al. reported elevated levels of seminal leukocytes were detected in patients with chronic prostatitis (Motrich et al., 2005). In our study, prostatitis was associated with higher odds of LCS. However, semen parameters were compared between men with and without LCS, not between men with and without prostatitis. Our results therefore do not resolve whether prostatitis directly affects individual semen parameters.

Epididymitis, characterized by acute swelling of the scrotum, is caused by *Chlamydia trachomatis* or *Neisseria gonorrhoeae* in sexually active young men and by *E. coli* in older men (Ludwig, 2008). The inflammation may spread to the testes, causing epididymo-orchitis, which is linked to high infertility rates (Osegbe, 1991). Chronic epididymitis has been associated with lower sperm counts and motility (Haidl et al., 2008), and changes in sperm protein composition have been reported several months after acute epididymitis (Pilatz et al., 2014). In particular, the present study suggests that epididymitis is associated with higher odds of LCS, supporting an association between epididymal inflammation and increased seminal leukocytes. Orchitis, an inflammatory lesion of the testes, is caused by similar pathogens in men under 35 and by *E. coli* in older men (Henkel et al., 2021). Orchitis results in tubular damage and testicular atrophy, leading to poor sperm quality and low sperm counts (Schuppe et al., 2008). Seminal vesiculitis, an inflammatory condition in the male genital tract, is often characterized by hematospermia (Liu et al., 2016). Its prevalence is lower than that of other male genital tract inflammations such as prostatitis, epididymitis, and orchitis (Xu et al., 2011). While seminal vesiculitis is often self-limiting, it can persist as a refractory condition that causes anxiety and may negatively impact male fertility (Mathers et al., 2017). However, the current study did not find a significant association between orchitis and seminal vesiculitis and LCS, possibly due to the low prevalence of these conditions, which may have limited the statistical power to detect such associations. Collectively, these results highlight the clinical relevance of evaluating epididymitis and prostatitis in male infertility, as the presence of LCS may indicate underlying inflammatory processes that may negatively impact sperm quality and fertility outcomes.

The current study exhibits several strengths. The large sample size allowed us to examine several clinical and lifestyle factors in men undergoing andrological evaluation. Semen analyses were performed using standardized laboratory procedures, and one semen-analysis record was retained for each participant. In addition, the multivariable models included prespecified demographic, lifestyle, and clinical variables, and the DAG was used to make the covariate structure clear.

However, several limitations must be carefully considered. First, the cross-sectional design does not establish the temporal sequence between urogenital inflammatory conditions and LCS. Reverse causation and differences in symptom reporting or clinical assessment are possible. Second, epididymitis, prostatitis, orchitis, and seminal vesiculitis were clinically defined and were not confirmed by pathogen-specific culture or nucleic-acid testing. Misclassification of these conditions may therefore have occurred, and the direction of any resulting bias cannot be determined. Third, although we retained the semen analysis linked to the questionnaire and clinical assessment, LCS was classified from a single ejaculate. Within-person biological and analytical variation in seminal leukocyte concentration may have led to misclassification, particularly around the 1 × 10^6^/mL threshold. Fourth, 3446 of the 12,791 initially screened records were excluded, mainly because of incomplete clinical information (n = 2903). Because most baseline and questionnaire variables were unavailable for these individuals, a comprehensive comparison between included and excluded participants could not be performed. Selection bias cannot be excluded if men with complete clinical information differed systematically from those with incomplete information. The analytical sample may therefore not fully represent all men attending the andrology clinic during the study period. Moreover, the cross-sectional design precludes establishing temporality, and thus the causal roles of chronic systemic diseases and varicocele could not be determined. Finally, residual confounding cannot be excluded, and the small numbers of men with orchitis or seminal vesiculitis limited the precision of the estimates for these conditions.

## Conclusions

In summary, our study found that epididymitis and prostatitis were associated with higher odds of LCS in men attending an andrology clinic. Men with LCS had lower semen volume, sperm concentration, progressive and total motility, TMSC, and normal morphology, and higher DFI and HDS. These findings are relevant to the evaluation of men with LCS and suboptimal semen quality, but they do not establish causality or support empirical antibiotic treatment in the absence of confirmed infection. Prospective studies with repeated semen analyses, microbiological testing, and detailed assessment of genital tract inflammation are needed.

## Data Availability

The original contributions presented in the study are included in the article/**Supplementary Material**. Further inquiries can be directed to the corresponding authors.

## Supplementary material

Supplementary Figure 1 Directed acyclic graph (DAG) of the hypothesized relationships between urogenital inflammatory conditions and LCS. Solid arrows represent the hypothesized relationships underlying Model 1. Dashed arrows represent clinically plausible relationships involving the additional covariates included in Model 2.

Supplementary Figure 2 Stratified multivariate logistic regression of the associations among epididymitis, prostatitis and LCS according to subgroup analysis.
